# Polling health-care workers opinion on assisted suicide and euthanasia: a word of caution

**DOI:** 10.1186/s13613-023-01139-4

**Published:** 2023-05-24

**Authors:** Nicolas de Prost, Marie Laurent

**Affiliations:** 1 Groupe de Recherche Clinique CARMAS, Université Paris Est-Créteil, Créteil, France; 2 Université Paris-Est Créteil Val de Marne (UPEC), Créteil, France; 3 Service de Médecine Intensive Réanimation, Hôpitaux Universitaires Henri Mondor– Albert Chenevier, Assistance Publique-Hôpitaux de Paris (AP-HP), Créteil, France; 4 Univ Paris Est Creteil, INSERM, IMRB, CEpiA Team, 94010 Creteil, France; 5 Service de Gériatrie, Hôpitaux Universitaires Henri Mondor-Albert Chenevier, Assistance Publique-Hôpitaux de Paris (AP-HP), Créteil, France

Dear Editor,

We read with interest the article by Acquier et al., reporting the results of a questionnaire self-administered to 1149 French intensive care unit (ICU) health-care workers (HCW) [1]. Although the French President Emmanuel Macron just released the results of a citizens’ convention on assisted dying, which supports active assistance in dying using assisted suicide and euthanasia [2], polling ICU HCW’s opinion about assisted dying might be particularly insightful in this context. The article of Acquier et al. concluded that “ *HCWs, particularly nonphysicians, would be in favor of a law legalizing euthanasia/physician-assisted suicide*”. We would like to highlight the following points, which we believe should be taken into consideration for the interpretation of the study results: (1) the age or ICU experience of the respondents were not recorded. The main results of the survey are driven by nonphysicians, mainly nurses, and more than 15% of physicians were medical residents. As such, analysis of the results by age classes or level of experience may have highlighted a relationship between experience and opinion on assisted suicide and euthanasia. These analyses could also have assessed the representativeness of the samples (less than 10% of the respondents). The choice of limiting this information to “*simplify the questionnary*” is surprising given the complexity of the current debate; (2) the authors did not address the respondents' familiarity with the Claeys−Leonetti law. Indeed, it is quite surprising to read that only 20.8% of the respondents replied that the current law « *almost always*» allows for managing end-of-life situations in ICU patients in France. Such result may be due to a lack of knowledge of the possibilities of the law, which typically applies to most of (if not all) the situations encountered in the ICU setting [3]; (3) Last, we fear that some of the items of the questionnaire might suffer from framing effects, when « *(often small) changes in the presentation of an issue or an event can produce (sometimes large) changes of opinion*» [4]. Importantly, framing effects have been previously shown to impact the results of assisted dying survey [5]. In the current case, both framing effects, including wording (e.g., questions 4, 5 and 6: « *would be desirable*»; « *would improve*») and context (e.g., question 4: « *a patient with major swallowing disorder refusing artificial nutrition*») effects may have given rise to a particular interpretation to the respondents, more likely to engage their sympathy towards euthanasia and physician-assisted suicide.

The results concerning opinions of ICU health-care physicians must be interpreted with caution. Further studies limiting the risk of selection biases associated with the HCWs must be interviewed (e.g., balancing the nonphysicians to physicians ratio, stratifying by age class) and taking into consideration the framing effects should be conducted.

## Data Availability

Non appropriate.

## Abbreviations

HCW: Health-care workers
ICU: Intensive care unit
